# Chitosan Nanoparticles Entrapping Aqueous *Psidium guajava* L. Leaf Extracts: A Promising Approach for Topically Treating Disorders in Oral Mucosa

**DOI:** 10.3390/plants14193099

**Published:** 2025-10-08

**Authors:** Renata Paula Coppini Almeida, Samuel Leite Cardoso, Seila Tolentino, Artur Fiuza Borges Arantes, Isabella Souza Mota, Guilherme Martins Gelfuso, Felipe Saldanha-Araujo, Eliete Neves Silva Guerra, Yanna Karla Medeiros Nobrega, Christopher William Fagg, Dâmaris Silveira, Yris Maria Fonseca-Bazzo, Pérola Oliveira Magalhães

**Affiliations:** 1 Laboratory of Natural Products, School of Health Sciences, University of Brasilia, Brasilia 70910-900, Brazil; coppinirenata@gmail.com (R.P.C.A.); samuca93@gmail.com (S.L.C.); arturborges_a@hotmail.com (A.F.B.A.); yannanobrega@unb.br (Y.K.M.N.); fagg@unb.br (C.W.F.); yrisfonseca@unb.br (Y.M.F.-B.); 2 Laboratory of Foods, Drugs, and Cosmetics (LTMAC), School of Health Sciences, University of Brasilia, Brasilia 70910-900, Brazil; seilatolentino@hotmail.com (S.T.); gmgelfuso@unb.br (G.M.G.); 3 Laboratory of Hematology and Stem Cells (LHCT), School of Health Sciences, University of Brasilia, Brasilia 70910-900, Brazil; isabellasmota11@gmail.com (I.S.M.); felipearaujo@unb.br (F.S.-A.); 4 Laboratory of Oral Histopathology, School of Health Sciences, University of Brasilia, Brasilia 70910-900, Brazil; elieteneves@unb.br

**Keywords:** *Psidium guajava* L., mucosal disorders, herbal medicine, mouthwash

## Abstract

*Psidium guajava* L. (Myrtaceae) is a neotropical species whose leaf extracts demonstrate efficacy against cutaneous and mucosal inflammation and ulceration. This study aimed to prepare and characterize aqueous extracts of *P. guajava* leaves (EAPG) and incorporate them into chitosan nanoparticles for topical delivery to the oral mucosa. The extract was obtained by infusion, and its marker compound was quantified by a chromatographic method. EAPG exhibited antioxidant activity (IC_50_: 6.35–7.01 µg/mL in DPPH^•^; FRAP: 14.42–17.83 µg/mL ≈ 60 µM Fe^2+^) and anti-inflammatory potential by modulating the expression of IL-6. It also showed antifungal activity against *Candida* species. Nanoparticles loaded with EAPG had a mean diameter of 899.8 ± 10.8 nm, PdI 0.22 ± 0.03, Zeta potential +32.4 ± 2.3 mV, pH 5.0, and 62 ± 1% encapsulation efficiency. They remained stable for 30 days. In an ex vivo topical application, EAPG nanoparticles delivered 415.17 ± 71.7 µg/cm^2^ of marker to the oral mucosa, eight times more than free EAPG (*p* < 0.05). These results suggest that chitosan-based EAPG nanoparticles are a promising strategy for topical treatment of mucosal disorders.

## 1. Introduction

*Psidium guajava* L. (Myrtaceae) is a neotropical species cultivated in South America and several other countries, such as India, Indonesia, and Pakistan [1]. The edible fruit, guava, is widely consumed and valued globally for both fresh fruit and processed products, such as beverages, purees, bakery goods, and dairy items like ice cream [2]. The global guava market size was valued at over USD 900 million in 2024, with projections estimating growth to USD 4.84 billion by 2033. Brazil ranks as the world’s fourth-largest exporter [3,4,5]. Beyond its economic relevance, *P. guajava* leaves hold recognized medicinal value, traditionally used in Brazilian folk medicine as teas to treat diarrhea and in topical applications for antiseptic purposes. Ethnopharmacological reports further highlight their use in treating inflammatory processes, local ulcers, oral and throat infections, and relieving toothache [1,6,7,8,9].

Inflammatory and ulcerative lesions of the oral mucosa are common clinical conditions that negatively impact oral health by causing pain, discomfort, and impaired oral function. Their etiology is diverse, ranging from infectious agents to immune-mediated disorders or mechanical trauma, and they are often associated with disruption of mucosal integrity and reduced quality of life [10,11]. These lesions may present as mild and self-limiting conditions or as severe disorders requiring complex therapeutic interventions and long-term follow-up. The distinction between these forms is crucial for effective management, informed treatment decision-making, and accurate prognosis. Less severe oral mucosal lesions, such as aphthous ulcers (canker sores) and traumatic or irritative injuries, are self-limiting and heal within a brief period, with or without treatment. They are commonly associated with accidental biting, aggressive toothbrushing, minor burns, or denture use [12]. In contrast, complex or persistent lesions often require specialized care and systemic therapy, as they may be linked to underlying systemic diseases. For example, large ulcerations can be associated with autoimmune disorders, such as oral lichen planus, while mucositis represents a severe inflammatory response of the mucosal epithelium to the cytotoxic effects of chemotherapy and radiotherapy [11,13].

Management of inflammatory and ulcerative oral mucosal lesions requires a personalized approach based on the underlying cause, severity, and overall clinical status of the patient. Therapeutic strategies aim to control inflammation, relieve symptoms, and promote healing, typically combining local and systemic treatments. While mild cases may benefit from antiseptic mouth rinses and topical corticosteroids, more severe conditions may demand systemic corticosteroids, antibiotics, or other pharmacological classes, which, despite their efficacy, carry the risk of toxicity and adverse effects [14,15].

To overcome the limitations associated with conventional treatments, particularly those related to toxicity or the potential for microbial resistance, the use of medicinal plants as adjuvants in the prevention and treatment of certain oral pathologies has gained increasing popularity, mainly due to their anti-inflammatory and antioxidant properties. Numerous studies have been conducted to validate the traditional use of medicinal plant species in managing these conditions [16].

In this context, numerous biological activities have been attributed to *Psidium guajava* extracts based on in vitro and in vivo studies, as well as clinical trials [17,18,19]. Both historical and recent medicinal applications of guava leaves highlight their therapeutic potential, extending beyond their conventional classification as a waste material.

Given the relevance of the traditional use of *Psidium guajava* extract as a potent antiseptic in the treatment of oral ulcers, this study aimed to prepare and characterize aqueous extract of *P. guajava* leaves for the management of mucosal disorders.

In the presented context, this study proposes the development of chitosan-based nanoparticles loaded with an aqueous extract of *Psidium guajava* leaves, offering significant advantages for topical applications. The nanoencapsulation approach provides enhanced stabilization of bioactive compounds and controlled release kinetics when applied to skin or mucous membranes [20]. Chitosan was selected as the ideal polymer matrix due to its natural abundance, excellent biocompatibility, and inherent mucoadhesiveness, which prolongs contact with mucosal surfaces while improving topical bioavailability. Importantly, certain nanoparticles have been well-documented as effective penetration enhancers, facilitating the transport of encapsulated molecules across epithelial barriers through multiple mechanisms: they modulate tight junction integrity, interact electrostatically with negatively charged mucosal surfaces [21], and create sustained local depots of active compounds.

Studies have demonstrated that light-controllable chitosan micelles loaded with thymol were effective in eliminating biofilms. Due to the presence of chitosan on their outer surface, thymol-loaded chitosan micelles readily bound to negatively charged biofilms through electrostatic interactions and efficiently released the essential oil cargo. Upon irradiation, these micelles generated reactive oxygen species (ROS), which not only triggered the simultaneous release of thymol but also induced additional ROS-mediated bactericidal effects, both of which effectively eradicated biofilms of *Listeria monocytogenes* and *Staphylococcus aureus* [22]. In another study, chitosan conjugated with streptomycin was shown to be more effective in eradicating established biofilms and killing biofilm-associated bacteria than streptomycin alone [23]. Furthermore, Zolfaghari et al. (2009) reported that the leaf extract of *Ocimum tenuiflorum* L., when loaded into chitosan nanoparticles as a drug carrier, exhibited significant anti-inflammatory activity [24]. Therefore, this delivery system strategically addresses both the pharmaceutical challenge of maintaining the stability of plant derivatives and the pharmacological need for controlled biodistribution.

In this study, an integrated phytopharmaceutical approach is employed, starting with the preparation and comprehensive characterization of *P. guajava* aqueous leaf extract (EAPG). This approach includes evaluating their antioxidant, antimicrobial, and anti-inflammatory properties through in vitro assays. Building on these pharmacological profiles, chitosan-based polymeric nanoparticles were engineered to encapsulate selected extracts, optimizing the formulation through systematic physicochemical characterization. The therapeutic potential of this delivery system was through ex vivo permeability studies using oral mucosa models, specifically tracking the penetration of extract biomarkers following topical nanoparticle application.

## 2. Results

### 2.1. Characterization of the Batches After the Extraction Process

The four extract batches demonstrated consistent but variable performance in terms of total solids content and extraction yields. Total solids quantification revealed values of 1.52 ± 0.25% (batch 1); 1.70 ± 0.20% (batch 2); 1.83 ± 0.15% (batch 3); and 1.70 ± 0.26% (batch 4). Corresponding extraction yields were 12.79%, 17.00%, 15.90%, and 15.67%, respectively. Moisture content analysis showed parallel variation across batches: 0.64 ± 0.06% (batch 1); 0.8 ± 0.05% (batch 2); 0.76 ± 0.06% (batch 3); and 0.80 ± 0.05% (batch 4). A clear correlation emerged between extraction yield and moisture retention, with batch 2 demonstrating both the highest yield (17.00%) and moisture content (0.85%). Conversely, batch 1 showed the lowest values for both parameters (12.79% yield, 0.64% moisture). This proportional relationship suggests that the extraction efficiency influenced the final product’s water content through differential retention of hydrophilic constituents during the concentration process. Quantification of total polyphenolic content revealed consistent values across all batches: 410.37 ± 2.54 mg GAE/g (batch 1); 453.23 ± 7.38 mg GAE/g (batch 2); 445.06 ± 5.10 mg GAE/g (batch 3); and 442.21 ± 7.87 mg GAE/g (batch 4). Statistical analysis (ANOVA, *p* < 0.05) confirmed no significant difference in polyphenolic content between batches.

**Figure 1:**
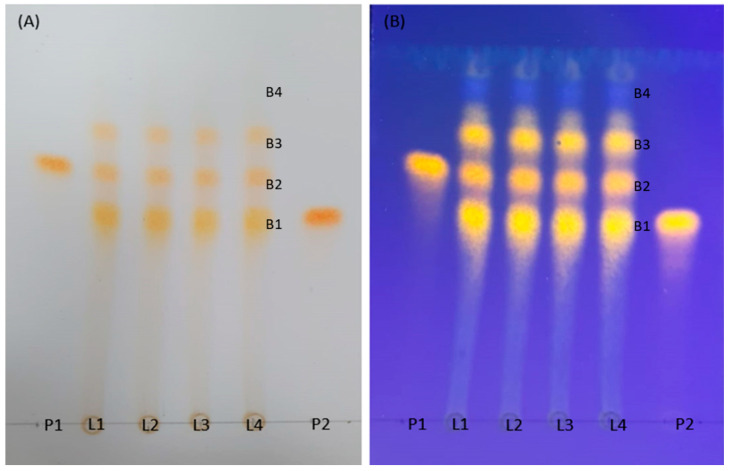
Thin-layer chromatography of the four batches (L1–L4) of *Psidium guajava* L. aqueous extracts (EAPG, 4.0 mg/mL) and the standards guaijaverin (P1) and hyperoside (P2) (1 mg/mL). Eluent: ethyl acetate-methyl alcohol-water-formic acid (20:2.7:2:0.2). (**A**) Chromatoplate (ALUGRAM^®^ SIL G Macherey-Nagel, Düren, Germany) revealed with NP-PEG; (**B**) Chromatoplate visualized under ultraviolet light (λ = 365 nm). B1–B4: Bands 1 to 4.

**Figure 2:**
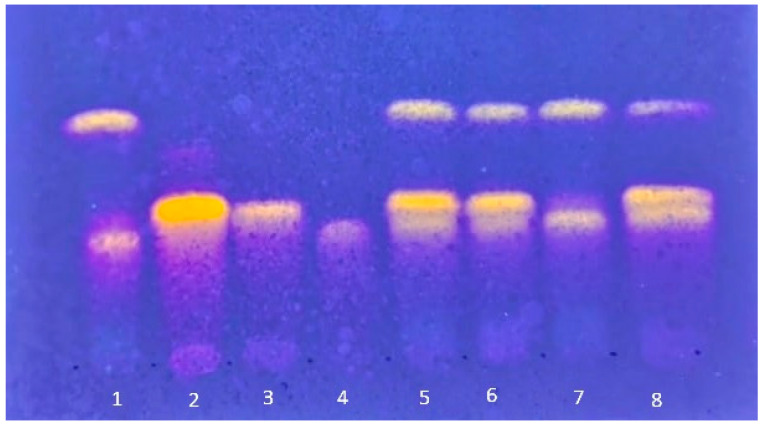
Thin-layer chromatography of batch L3 of *Psidium guajava* L. aqueous extracts (EAPG) and standards. Eluent: acetonitrile: formic acid (1%) (40:60). Chromatoplate (TLC Silica gel 60 RP-18 F_254_ Supelco, Bellefonte, PA, USA) revealed with NP-PEG and visualized under ultraviolet light (λ = 365 nm). 1: EAPG; 2: hyperoside; 3: isoquercitrin; 4: guaijaverin; 5: EAPG-hyperoside co-elution; 6: EAPG-isoquercitrin co-elution; 7: EAPG-guaijaverin co-elution; 8: EAPG-hyperoside-isoquercitrin-guaijaverin co-elution.

Complementary phytochemical analysis by Thin-Layer Chromatography (TLC) confirmed the reproducibility of the extraction process. As shown in Figure 1, all four evaluated batches yielded nearly identical chromatographic profiles, demonstrating consistent phytochemical composition across the samples. To verify the presence of key compounds previously reported in *P. guajava* leaves—guaijaverin, hyperoside, and isoquercitrin—a subsequent TLC analysis was performed. This experiment involved co-eluting the extract with each reference standard individually, as well as with a mixture of all three standards (Figure 2).

Based on the TLC analysis presented in Figure 2, it can be inferred that, even using the co-elution technique, a conclusive confirmation regarding the presence of guaijaverin, hyperoside, and isoquercitrin was not possible, probably due to the very close similarity of the target compounds.

**Figure 3:**
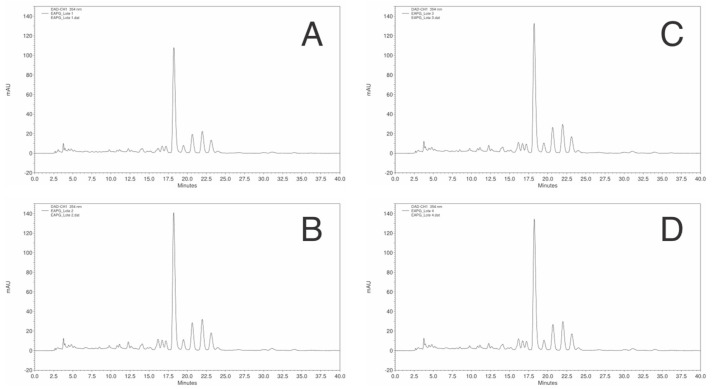
Chromatogram obtained by HPLC-DAD of *Psidium guajava* L. aqueous extracts (EAPG, 5 mg/mL). Analysis conditions: PurospherStar RP C18e (Merck, Darmstadt, Germany) column maintained at 25 °C; eluent: solvent A (1% phosphoric acid), solvent B (acetonitrile); detector: DAD; elution system: gradient; flow rate 0.6 mL/min; (**A**) EAPG batch 1; (**B**) EAPG batch 2; (**C**) EAPG batch 3; (**D**) EAPG batch 4.

Moreover, the high-performance liquid chromatography analysis confirmed the robustness of the extraction protocol, showing a remarkable similarity in both quantitative polyphenolic content and qualitative chromatographic profiles across four independent extractions (Figure 3).

**Figure 4:**
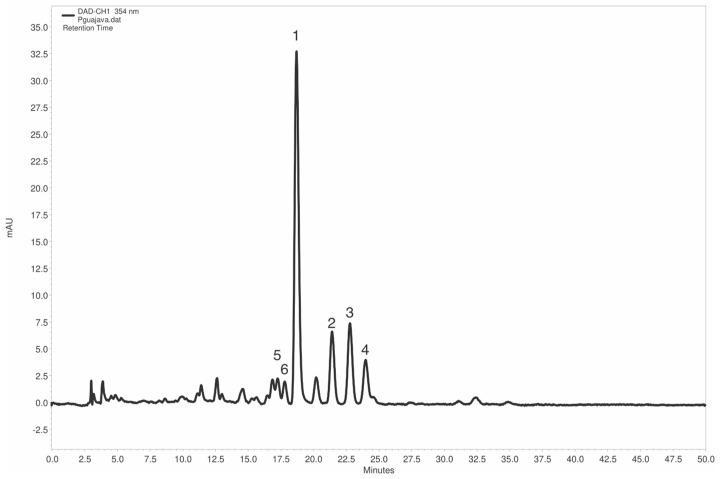
Representative HPLC chromatograms of *Psidium guajava* L. aqueous extracts (EAPG, 5 mg/mL). Chromatographic conditions: C18 column PurospherStar RP C18e column (150 × 4.6 mm, 5 mm, (Merck, Darmstadt, Germany). The detector was set to collect data at 354 nm. The mobile phase consisted of a 1% phosphoric acid and acetonitrile gradient at a flow rate of 0.6 mL/min, starting at 85% (1% phosphoric acid), decreasing to 75% until t = 45 min, and then returning to 85% until t = 50 min. The sample injection was 10 µL. All solvents used as mobile phase were filtered through a 0.22 μm, 47 mm polyvinylidene fluoride (PVDF) membrane (Merck, Darmstadt, Germany,).

Supplementing the relevant information on EAPG and the extraction process, Figure 4 presents a representative chromatogram of EAPG, in which four prominent, well-resolved peaks (labeled 1–4) with adequate purity are observed, besides others that are not well resolved (e.g., 5 and 6). However, in a first instance, it was not possible to identify any compound with a minimum similarity of 0.998 in the UV spectra or with the same retention time as the tested standards (myricetin, guaijaverin, caffeic acid, chlorogenic acid, ferulic acid, kaempferol, rosmarinic acid, hesperetin, hesperidin, ellagic acid, gallic acid, hyperoside, quercetin, isoquercitrin, resveratrol, rutin, vitexin, and isovitexin).

**Figure 5:**
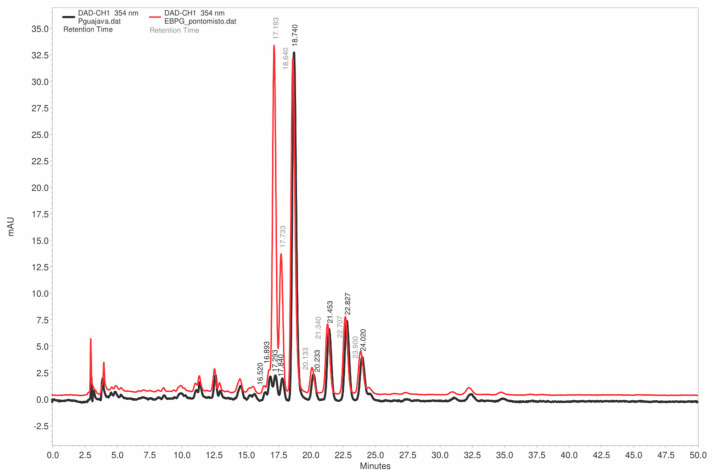
Overlay of representative HPLC chromatograms of *Psidium guajava* L. aqueous extracts (EAPG, black line) and *Psidium guajava* L. aqueous extracts enriched with isoquercitrin, hyperoside, and guaijaverin standards (red line). Chromatographic conditions: C18 column PurospherStar RP C18e column (150 × 4.6 mm, 5 mm, Merck, Darmstadt, Germany). The detector was set to collect data at 354 nm. The mobile phase consisted of a 1% phosphoric acid and acetonitrile gradient at a flow rate of 0.6 mL/min, starting at 85% (1% phosphoric acid), decreasing to 75% until t = 45 min, and then returning to 85% until t = 50 min. The sample injection was 10 µL. All solvents used as mobile phase were filtered through a 0.22 μm, 47 mm polyvinylidene fluoride (PVDF) membrane (Merck, Darmstadt, Germany).

Based on the results of the TLC analyses (Figure 2) and the high UV spectral similarity (0.9962) observed between the main peak of EAPG (retention time 18.7 min) and the isoquercitrin standard (17.2 min) (Figure 4), an additional HPLC-DAD study was performed using EAPG samples individually enriched with isoquercitrin, hyperoside, and guaijaverin standards (Figure 5).

Upon enrichment with guaijaverin, a slight increase in the peak area (18.77%) was observed at a retention time of 18.7 min. The UV spectrum of this peak demonstrated alignment with the guaijaverin standard, yielding a similarity score of 0.9974. Correspondingly, enrichment with hyperoside and isoquercitrin standards resulted in increased peak areas for minor constituents at 17.2 min and 17.7 min, respectively. The UV spectra corresponding to these peaks showed alignment with their respective standards, though the calculated similarity scores fell slightly below the confirmation threshold of 0.998. Consequently, while the chromatographic and spectral data provide supportive evidence, a definitive identification of the compounds corresponding to peaks labeled 1, 5, and 6 (Figure 4) cannot be asserted without further structural elucidation. The isolation and comprehensive characterization of these specific constituents have therefore been designated as our future research focus.

### 2.2. In Vitro Cytotoxicity Assay

**Figure 6:**
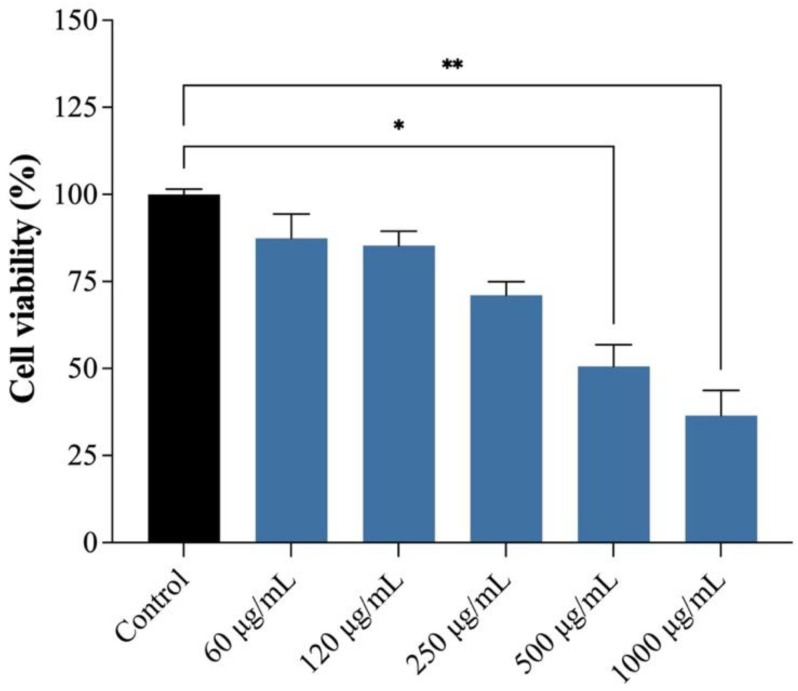
Effect of *Psidium guajava* L. aqueous extract (EAPG) on cell viability of gingival fibroblast (hGF) cells under EAPG treatment. The MTT assay was performed to measure the viability of hGF after 48 h of treatment with increasing concentrations of EAPG. The control group is represented by untreated cells. Results are presented as mean ± SD. The statistical analysis (Kruskal–Wallis) compares the treatment with the control (* *p* < 0.05; ** *p* < 0.05).

The therapeutic benefits of products derived from plant species are indisputable, which has driven their incorporation into various clinical products. However, it should be accompanied by an understanding of its effect on different cell lines to ensure the safe use of these products [25]. In this context, the analysis of plant extracts’ cytotoxicity enables the identification of potential cellular degeneration or death induced by them, demonstrating how plant-derived substances can harm various cells of interest. The cytotoxicity assessment of EAPG in human gingival fibroblasts (hGFs) revealed concentration-dependent effects. While the cells maintained tolerance at lower concentrations during the 48 h exposure, a statistically significant reduction in viability occurred at concentrations of 500 µg/mL or higher (Figure 6). The concentration of EAPG required to inhibit 50% of cell growth (IC_50_) was 709.6 µg/mL. The results align with previous findings reporting the cytotoxic activity of a *P. guajava* hydroalcoholic extract (70% ethanol) in human gingival fibroblasts [26].

Previous studies demonstrated concentration-dependent toxicity patterns for *P. guajava* extracts across various cell lines. Millones-Gómez et al. (2022) reported high cell viability (99.7 ± 1.24% in HeLa; 99.8 ± 2.2% in HGF-1; and 99.7 ± 2.7% in PBMC) at 0.24 mg/mL of an ethanolic extract obtained from *P. guajava* fruits [25]. Similar non-cytotoxic effects were observed for methanolic leaf extracts (100 µg/mL) and their fractions (hexane, dichloromethane, ethyl acetate, n-butanol, and aqueous) in the RAW264.7 macrophage cell line, comparable to untreated controls. Notably, ursolic acid (10 µM), isolated from a methanolic extract, also maintained the cell viability [27,28]. These findings collectively suggest that *P. guajava* derivatives exhibit favorable safety profiles at low concentrations [25,29,30], supporting their ethnopharmacological use as oral antiseptics for over two decades [1]. The concentration-dependent response observed in these studies aligns with the current findings, where cytotoxicity only emerged at higher concentrations (≥500 μg/mL). This substantial pre-clinical evidence validates the therapeutic potential of *P. guajava* while highlighting the critical importance of dose optimization. The established safety window at lower concentrations provides a foundation for developing standardized phytopharmaceutical formulations, particularly for oral mucosal applications where traditional use has been well-documented.

### 2.3. Antioxidant Activity Assay

Reactive oxygen species (ROS) have been identified as critical mediators in the pathogenesis of oral diseases, contributing to both direct tissue damage and the perpetuation of hyperinflammation states through activation of key nuclear transcription factors, such as nuclear factor kappa B (NF-kB) and activator protein-1 (AP-1), which regulate expression of major proinflammatory mediators [11]. Therapeutic strategies targeting oxidative stress through the use of antioxidant compounds, particularly plant-derived compounds such as quercetin and ferulic acid, may help mitigate oxidative damage while modulating cytokine production [28,31,32]. To comprehensively evaluate this potential, the antioxidant activity of both EAPG and its chitosan microparticles formulation was assessed using two well-established methods: the 2,2-diphenyl-1-picrylhydrazyl (DPPH^•^) radical scavenging activity assay to determine free radical-neutralizing capacity, and the Ferric Reducing Antioxidant Power (FRAP) assay to measure reducing power, thereby providing complementary insights into their redox-modulating properties relevant for oral health applications.

Both EAPG and its chitosan microparticle formulation were evaluated at identical concentrations (3.9 to 250 µg/mL) in parallel with placebo particles to account for excipient interference. The DPPH^•^ radical scavenging assay, using ascorbic acid (0.39 to 25 µg/mL) as a positive control, showed potent antioxidant activity for both preparations. At the maximum tested concentration (250 µg/mL), EAPG exhibited 89.99% radical scavenging capacity while the loaded particle solution showed comparable efficacy (93.57%). Quantitative analysis revealed an IC_50_ of 7.35 μg/mL for EAPG versus 3.13 μg/mL for ascorbic acid, confirming the extract’s significant antioxidant potential.

To complement the DPPH assay findings and address its limitations regarding biological relevance [33], the Ferric Reducing Antioxidant Power (FRAP) assay was conducted across a concentration range of 1.83 to 29.41 µg/mL for both EAPG and its microparticle formulation, alongside placebo controls (showing no activity) and ascorbic acid (0.31–10 µg/mL). The results were expressed as the sample concentration equivalent to 60 µM of reduced iron (Fe^2+^). The use of a second assay to evaluate the antioxidant activity of EAPG was essential, as the DPPH^•^ assay has some significant limitations, including the fact that the radical is not naturally found in biological systems and does not resemble the more reactive free radicals [33].

The FRAP assay results demonstrate the superior antioxidant capacity of EAPG, with only 14.42 µg/mL required to achieve an antioxidant effect equivalent to 60 µM of reduced iron (Fe^2+^). This result surpassed that of chitosan microparticle formulation (17.83 µg/mL needed for equivalent activity) and approached the potency of ascorbic acid (5.0 μg/mL of ascorbic acid was equivalent to 60 µM of Fe^2+^). The extract’s reduction power can be attributed, at least in part, to its high content of phenolic compounds, which are known for their effective electron-donating capabilities [34]. These findings corroborate previous studies demonstrating the strong antioxidant potential of *P. guajava* [33] and support its therapeutic application for oxidative stress-related conditions. The slight reduction in activity observed in the formulation may reflect minor interactions between the extract’s bioactive components and the chitosan matrix during encapsulation; however, the overall antioxidant capacity remained well-preserved.

### 2.4. EAPG Inhibits the Expression of the Proinflammatory Cytokine IL-6

**Figure 7:**
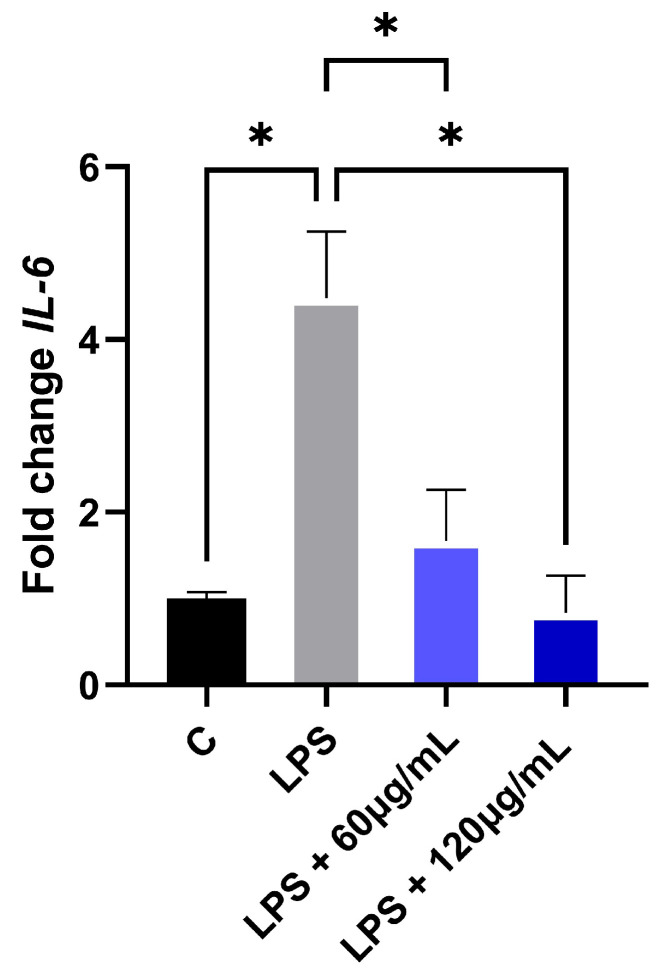
Gene expression by RT-qPCR. *Psidium guajava* L. aqueous extract (EAPG) inhibits the production of IL-6 by hGFC after their exposure to lipopolysaccharide (LPS). The fold changes were determined by the 2^−ΔΔCt^ method, using the median Ct value of control hGFC as a reference. The control group is represented by untreated cells (C). Results are presented as mean ± SD. The statistical analysis (Kruskal–Wallis) compares the treatment with the control (* *p* < 0.05).

The study of oral inflammatory disorders has gained significant momentum as researchers investigate the interplay between genetic predisposition and environmental triggers in mucosal pathogenesis [11]. The evaluation of EAPG revealed potent immunomodulatory effects in human gingival fibroblast (hGF) cells. Lipopolysaccharide (LPS) stimulation induced a 4.5-fold upregulation of the proinflammatory cytokine IL-6 expression, establishing a robust inflammatory model. Notably, treatment with EAPG at both 60 and 120 μg/mL significantly attenuated this response, demonstrating dose-dependent suppression of IL-6 secretion (Figure 7). These findings suggest that EAPG contains compounds capable of modulating key inflammatory pathways in oral mucosal tissues, potentially through interference with the LPS-induced signaling cascade.

Interleukin-6 (IL-6) serves as a master of both physiological and pathological processes, including chronic inflammatory, autoimmune, infectious, and neoplastic diseases [31]. In oral health, IL-6 has emerged as a key mediator in the development of mucosal lesions and the progression of periodontitis, with its levels serving as both diagnostic and prognostic indicators [30]. Bacterial infection, particularly through LPS stimulation, potently induces IL-6 secretion [35], making this cytokine a valuable molecular marker for oral infection [36]. The pleiotropic effects of IL-6 are mediated through three distinct signaling pathways (classical, trans-signaling, and trans-presentation), all of which converge on JAK/STAT activation. This multifaceted cytokine bridges innate and adaptive immunity by serving as the primary inducer of acute-phase reactants, modulating T and B lymphocyte responses, and regulating Th17/Treg balance in mucosal immunity [31]. In this context, *P. guajava* has demonstrated remarkable therapeutic potential as a natural anti-inflammatory agent. According to the present findings, EAPG was able to suppress LPS-stimulated gingival fibroblasts, corroborating its traditional use.

The anti-inflammatory efficacy of *P. guajava* leaf extracts has been well-documented across multiple experimental models. Ghaderi et al. (2022) demonstrated that a hydroalcoholic extract significantly reduced serum IL-6 levels in rats with induced oral mucositis, as confirmed through phytochemical, biochemical, histopathological, and stereological studies [33]. These findings align with earlier work by Peng et al. (2011), which showed dose-dependent suppression of IL-6 expression (<1 mg/mL) in human prostate carcinoma cells treated with an aqueous polyphenolic fraction of *P. guajava* leaves [37]. This activity appears to stem from *P. guajava* rich polyphenolic composition, particularly flavonoids derived from quercetin, which modulate key inflammatory mediators [38]. Biological data have shown that the plant species exhibits significant anti-inflammatory activity against acute, subacute, and chronic inflammation, as well as a promising decrease in iNOS, COX-2, and NF-κB [39,40].

### 2.5. Antimicrobial Activity

**Figure 8:**
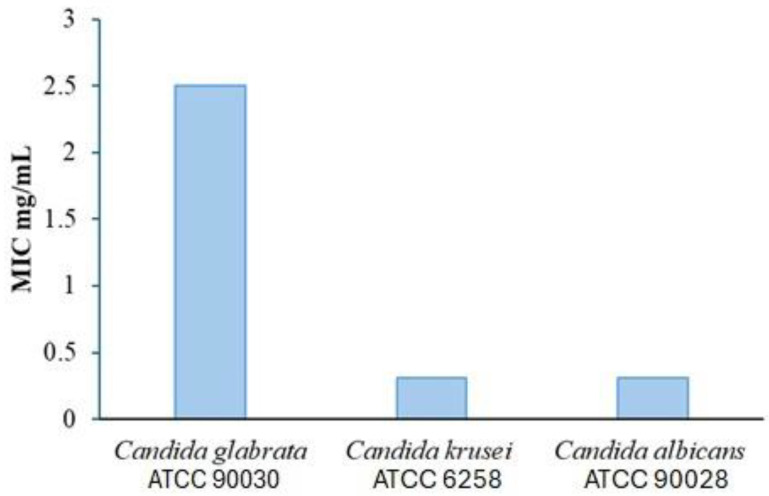
Minimal Inhibition Concentration (MIC) of *Psidium guajava* L. aqueous extract (EAPG) against *Candida glabrata* ATCC 90030, *Candida krusei* ATCC 6258, and *Candida albicans* ATCC 90028 strains.

The EAPG exhibited antifungal activity against clinically relevant *Candida* species, with minimal inhibitory concentration (MIC) values ranging from 0.312 to 2.5 mg/mL (Figure 8). Notably, *Candida albicans*, the most prevalent fungal pathogen in oral infections, exhibited the highest sensitivity to the extract. These results complement existing research supporting the therapeutic application of *P. guajava* leaf preparations against oral pathogens, including microorganisms associated with gingivitis, and contribute to other studies that have demonstrated the applications of this species against agents that cause gingivitis [41].

The antimicrobial potential of *P. guajava* leaf extracts has been demonstrated across multiple studies using various extraction methods. While EAPG showed activity against three *Candida* species, previous research reported high activity for certain preparations. Essential oil from *P. guajava* leaves exhibited antimicrobial activity against key oral pathogens, including *C. albicans* and *Streptococcus mutans*. The crude methanolic extract of P. guajava was also found to be promising against fluconazole-resistant strains of *C. albicans* [42].

### 2.6. Preparation and Characterization of the EAPG Nanoparticles

In this study, chitosan nanoparticles encapsulating EAPG were developed through a single-step, organic solvent-free process to, in a first approach, enhance the herbal derivative stability, facilitating their use as a pharmaceutical ingredient. The nanoparticles exhibited optimal characteristics, with a mean diameter of 899.8 ± 10.8 nm (all <1 µm in size), and low poly dispersibility (PdI of 0.22 ± 0.03), indicating a monomodal and homogeneous population suitable for topical applications, as it prevents aggregate formation, which could impair dose uniformity and cause sensory issues during application while ensuring a good mass/area ratio, providing good interaction between the nanomaterial and the mucus [43,44]. The formulation’s strong positive Zeta potential (+31.7 ± 2.0 mV), attributed to chitosan’s amino groups, ensures both excellent colloidal stability (Zeta potential > 30 mV) and mucoadhesive properties through electrostatic interactions with negatively charged mucosal surfaces [45,46,47,48]. These features promote prolonged residence time, previously evidenced by our group [21]. Other authors demonstrated that such a polymeric nanoparticle product adhered to the oral mucosa for 8 h without irritation [49,50].

**Figure 9:**
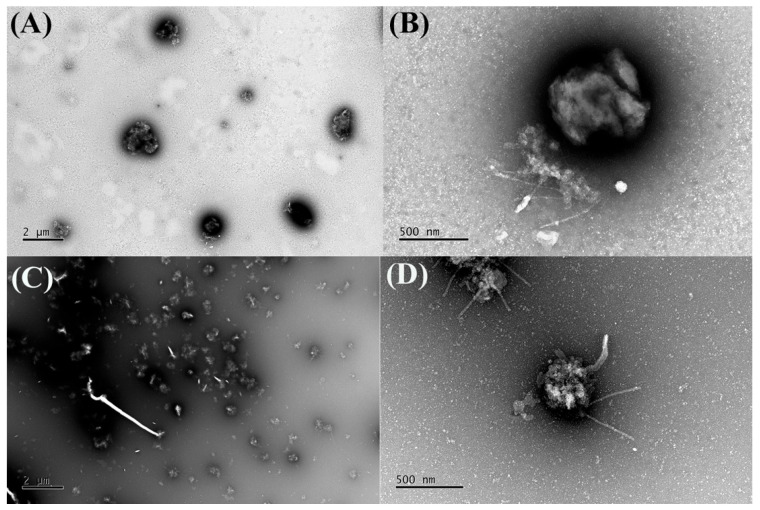
Representative photomicrograph of chitosan nanoparticles unloading (**A**,**B**) and loading *Psidium guajava* L. aqueous extracts (EAPG) (**C**,**D**) obtained by transmission electron microscopy (TEM JEM-1011, JEOL, Tokyo, Japan) at 5000× and 20,000× magnifications.

Morphological analysis, as observed by TEM, confirmed the presence of predominantly spherical chitosan nanoparticles with diameters exceeding 500 nm (Figure 9), exhibiting a monodisperse size distribution. The size and shape of the chitosan nanoparticles were not modified with the presence (Figure 9C,D) or absence (Figure 9A,B) of the EAPG.

The chitosan nanoparticles demonstrated high encapsulation (62 ± 1%) for the evaluated marker from the EAPG (peak 1). This substantial loading capacity can be attributed, at least in part, to the molecular affinity between certain phenolic compounds present in the EAPG extract and the chitosan matrix [50], facilitating hydrogen bonding and polar interactions among them.

### 2.7. Stability Studies

This study aimed to predetermine the shelf life of the aqueous suspension of nanoparticles loaded with EAPG and evaluate the best storage conditions. Table 1 and Table 2 present the observed results related to the assessed periods during the stability tests of the samples.

Our studies demonstrated that, regardless of the storage temperature, the chitosan nanoparticle formulation exhibited excellent physicochemical characteristics, which remained unchanged for 30 days, with no significant alterations in nanoparticle aggregation or the amount of encapsulated marker. Crucially, the formulation preserved a pH range (4.9–5.0) compatible with both chitosan stability and oral mucosal tolerance [51]. While refrigerated samples maintained optimal organoleptic properties, room temperature storage led to increased opacity, likely indicating extract leakage and eventual microbial contamination by the study endpoint. This can be attributed to the amount of extract outside the nanoparticles and indicates two critical formulation requirements for the final product: (i) incorporation of antioxidant additives to prevent extract degradation and nanoparticles destabilization; and (ii) addition of antimicrobial preservatives to enable room temperature storage.

### 2.8. Ex Vivo Mucosal Penetration Assay

Ex vivo penetration assays demonstrated that chitosan nanoparticles significantly enhanced the localized delivery of EAPG compared with the control solution. After 12 h of application, the chitosan nanoparticle formulation promoted EAPG penetration at different concentrations. It achieved mucosal retention of 415.17 ± 71.7 µg/cm^2^, which was eightfold higher (*p* < 0.05) than that of the control (51.38 ± 19.2 µg/cm^2^), confirming its superior penetration efficiency. Importantly, EAPG was undetectable in the receptor medium for both formulations, indicating minimal systemic permeation, a desirable characteristic for topical therapies that aim for localized effects without systemic absorption, as confirmed by the analytical method. The significant retention of EAPG in the mucosa supports the evaluation of topical formulations in terms of their ability to deliver active ingredients into different mucosal layers during formulation development. Moreover, it allows the assessment of whether the compound has the potential to reach systemic circulation.

Altogether, these findings highlight chitosan nanoparticles as an effective delivery system for enhancing mucosal retention while restricting systemic permeation, making them suitable for localized therapeutic applications.

## 3. Materials and Methods

### 3.1. Chemicals and Reagents

Guaijaverin (purity > 99%), hyperoside (purity > 99%), isoquercitrin (purity > 99%), Dulbecco’s modified eagle medium (DMEM), mineral oil, chitosan of medium molecular weight (190–310 kDa), ampicillin, amoxicillin, (3,[4,5-dimethylthiazol-2-yl]-2,5-diphenyltetrazolium bromide) (MTT), sodium tripolyphosphate (STPP), low molecular weight chitosan, and acetic acid were purchased from Sigma-Aldrich (Sigma-Aldrich, St. Louis, MO, USA). Dibasic sodium phosphate, sodium phosphate monohydrate, and 2,2′,2″,2‴-(ethane-1,2-diyldinitrilo) tetraacetic acid (EDTA) were obtained from Vetec (Vetec, Rio de Janeiro, Brazil). High-performance liquid chromatography (HPLC)-grade solvents, including phosphoric acid, methanol, and acetonitrile, were acquired from Tedia (Tedia, Fairfield, CT, USA). Ultra-purified water was prepared using a Milli-Q system (Merck KGaA, Darmstadt, Germany). 

### 3.2. Plant Material

Leaves of *Psidium guajava* L. were identified and collected on 25 June 2019 in Brasilia, Federal District, Brazil. Professor Christopher William Fagg performed the botanical identification, and a voucher specimen (Fagg CW2557) was deposited in the Herbarium of the University of Brasília (UB) for permanent record. This research was properly registered in the Brazilian National System of Management of Genetic Heritage and Associated Traditional Knowledge (SISGEN) under accession number A215A9A, in compliance with national regulations regarding biodiversity research.

### 3.3. Plant Material Processing and Extraction Protocol

Following collection, the leaves underwent controlled dehydration to meet the requirements of the Brazilian National Health Surveillance Agency (Anvisa) for the registration of herbal medicines [52]. The drying process was carried out using two complementary methods: (i) natural drying (at room temperature, in the shade, for 24 h), followed by (ii) oven drying [SOLAB (Piracicaba, Brazil), model SL–102], at 37 °C, until the target moisture content of 12% was reached. The dried leaves were then uniformly powdered using a Marconi MA-580^®^ mill (Caxias do Sul, Brazil) (30 mesh sieve) and aliquoted into four 100 g batches.

The aqueous extracts were prepared by infusion at a 1:10 (*w*/*v*) ratio, using distilled water as the solvent. Briefly, the powdered plant material was placed in an Erlenmeyer flask, and hot water was poured over it. The flask was covered and left to stand until it cooled to approximately 50 °C (10–20 min). The infusion was then filtered, and the extractive solutions were immediately frozen and subjected to the lyophilization process in an SP Scientific Advantage Plus XL-70 freeze-dryer (Cambridge Scientific, Watertown, MA, USA), under the following conditions: −70 °C condenser temperature and 15 mTorr vacuum pressure maintained for 14 days. The resulting lyophilized powder was stored at −20 °C until used for evaluations regarding the reproducibility of the extraction process, total solids content, yield, moisture content, total polyphenol content, antioxidant activity evaluation, and chromatographic fingerprint acquisition.

### 3.4. Preparation of Standard Solution

Stock solutions of standards (guaijaverin, hyperoside, and isoquercitrin) were prepared at a concentration of 1.0 mg/mL in methanol using a volumetric flask to ensure precision. The solution was stored under refrigeration (4 °C) and remained stable for up to one month before use.

### 3.5. Preparation of EAPG Sample Solutions

Aqueous extract of *P. guajava* leaves (EAPG) was weighed (20 mg) and solubilized in water (4 mL) to a final concentration of 5 mg/mL. All extract samples were filtered through a 0.45 μm filter before analysis.

### 3.6. Chromatographic Analyses

#### 3.6.1. Thin-Layer Chromatography (TLC)

The chemical profile of the lyophilized *P. guajava* leaf extracts (EAPG) was analyzed by thin-layer chromatography (TLC) following an optimized protocol based on modification of a previously described method [53]. Briefly, 10 mg of each extract was accurately weighed Shimadzu AY220 analytical balance (Shimadzu, Kyoto, Japan) and dissolved in 2.5 mL of distilled water (4 mg/mL). Chromatographic separation was performed on pre-coated silica gel 60 F_254_ aluminum plates (Machery-Nagel, Düren, Germany) using a mobile phase of ethyl acetate, methanol, water, and formic acid (20:2.7:2:0.2 *v*/*v*/*v*/*v*). After development, plates were derivatized with NP-PEG reagent (1% diphenylborioxethylamine + 5% polyethylene glycol 400), purchased from Sigma-Aldrich, São Paulo, Brazil, and visualized under ultraviolet (UV) light (λ = 365 nm) in a UV chamber (Prodicil, Pinhais, Brazil) for enhanced detection of phenolic compounds. For compound identification, samples were compared against reference standards (1 mg/mL each). Retention factors (Rf) were calculated for all resolved bands, allowing for a comparative analysis of the migration patterns between the extract and authentic standards. The TLC profiles obtained for each sample were compared with those of the others.

A subsequent TLC analysis was conducted using pre-coated silica gel 60 RP-18 F_254_ aluminum plates (Sulpelco, Bellefonte, PA, USA), with a mobile phase of acetonitrile and 1% aqueous formic acid (4:6 *v*/*v*). This experiment involved the co-elution of EAPG samples individually with guaijaverin, hyperoside, and isoquercitrin standards to verify the presence of compounds. Derivatization and visualization were performed as described above, using NP-PEG reagent and UV detection at 365 nm.

#### 3.6.2. High-Performance Liquid Chromatography Coupled to a Diode Array Detector (HPLC-DAD)

The chemical profile of the lyophilized *P. guajava* leaf extracts (EAPG) was characterized using an HPLC-DAD method adapted from a previously established protocol [54]. Briefly, analyses were performed on a Hitachi LaChrom Elite System (Hitachi, Tokyo, Japan) equipped with L2130 pump, L2200 injector, column oven L2300, and L2455 diode array detector. Separation was achieved using a C18 Purospher Star column (150 × 4.6 mm, 5 mm, Merck, Darmstadt, Germany) protected by a matching pre-column (4 × 4; 5 mm, Merck, Darmstadt, Germany) maintained at 25 °C. The mobile phase consisted of a gradient of 1% phosphoric acid (A) and acetonitrile (B) at a flow rate of 0.6 mL/min, programmed as follows: initial conditions of 85% A, decreasing to 75% A over 45 min, followed by a return to initial conditions by 50 min. Detection was performed at 354 nm, with an injection volume of 10 µL. All solvents were filtered through a 0.22 μm, 47 mm polyvinylidene fluoride (PVDF) membrane (Merck, Darmstadt, Germany) before use. Data acquisition and processing were conducted using EZChrom Elite software (version 3.3.2 SP1, Scientific Software Inc., Lincolnwood, IL, USA).

Following the initial fingerprint, target compound identification was performed by enriching samples of EAPG (4 mg/mL) with individual standards of hyperoside, isoquercitrin, and guaijaverin, as well as a combined standard mixture. Enriched samples were reanalyzed to observe changes in peak areas and retention times, thereby confirming candidate compounds based on co-elution and spectral matching.

### 3.7. Extract Characterization

#### 3.7.1. Determination of Total Solids Content

The total solids content of EAPG was determined in triplicate using 2 mL aliquots of the filtered extract. Measurements were performed using an infrared moisture analyzer (Gehaka IV2000, São Paulo, Brazil) preheated to 100 °C and operated in auto-dry mode.

#### 3.7.2. Calculation of the Extraction Yield

The extraction yield of EAPG was determined gravimetrically and expressed as a percentage of the total mass. The lyophilized extract mass was divided by the initial mass of the powdered leaves used in the extraction process.

#### 3.7.3. Determination of EAPG Moisture Content

The residual moisture content of EAPG was determined in triplicate using an infrared moisture analyser (Gehaka IV2000, São Paulo, Brazil). Samples (approximately 3.0 g) from each batch were evenly distributed in the analyzer chamber, which was preheated to 100 °C and operated in auto-dry mode. The moisture content was calculated based on weight loss during drying.

#### 3.7.4. Determination of Total Polyphenol Content

The total polyphenol content of EAPG was quantified using an adapted Folin–Ciocalteu colorimetric method, as described by Kumazawa et al. (2004) [55]. An analytical standard curve was prepared using a gallic acid stock solution (1 mg/mL in distilled water) at concentrations ranging from 3.33 μg/mL to 46.66 μg/mL. All samples were analyzed in triplicate with blank corrections, and the results were expressed as mg gallic acid equivalent (GAE) per gram of dry extract.

### 3.8. Gene Expression in Primary Culture of Human Gingival Fibroblast Cells (hGFC)

All study procedures were conducted in accordance with the Declaration of Helsinki and were approved by the Research Ethics Committee of the Faculty of Health Sciences, University of Brasilia (CAAE: 35371514.5.0000.0030). As previously described [56], primary human gingival fibroblast cells (hGFC) were isolated from gingival tissue obtained during third molar extractions from five healthy donors (aged 18–23 years). Tissue samples were processed by washing three times with phosphate-buffered saline (PBS), followed by mincing into fragments of 1–2 mm^3^. These explants were cultured in growth medium consisting of Dulbecco’s modified Eagle’s medium (DMEM) supplemented with 20% fetal bovine serum (FBS), antibiotics (10,000 IU/mL penicillin G sodium, 100,000 μg/mL streptomycin sulfate, 25 μg/mL amphotericin B), and 1% L-glutamine, maintained at 37 °C, in a 5% CO_2_ atmosphere. The culture medium was refreshed every four days during cell expansion. Cells between passages 3 and 6 were used for all experiments, following trypsin-EDTA harvesting.

To assess the anti-inflammatory potential of EAPG, an LPS-induced inflammation model was used in hGF cells. Cells were plated at 6 × 10^4^ cells/well in 12-well plates and stimulated with 1 μg/mL of LPS (lipopolysaccharide of *Escherichia coli* 0111: B4, Sigma-Aldrich, St. Louis, MO, USA) to induce an inflammatory environment. Immediately after LPS challenge, cells were treated with either 0.06 or 0.12 mg/mL of EAPG for 48 h, with untreated hGF serving as control. Following treatment, total RNA was extracted using TRI Reagent (Sigma-Aldrich, St. Louis, MO, USA) according to the manufacturer’s protocols. After RNA extraction, its concentration and purity were assessed, and 1 μg of total RNA was reverse-transcribed into cDNA using the High-Capacity cDNA reverse transcription kit (Thermo Fisher, Waltham, MA, USA).

IL-6 expression levels were determined by RT-qPCR (QuantStudio 1 Real-Time PCR System (Applied Biosystems, CA, USA). The reactions were performed in duplicate, using the SYBR Green method (Thermo Fisher) combined with specific primers (forward: 5′-TCAATATTAGAGTCTCAACCCCCA-3′; reverse: 5′-TTCTCTTTCGTTCCCGGTGG-3′). Relative fold value was obtained by the 2^−ΔΔCt^ method, and the median Ct values of the samples from untreated cells (control group) were used as a reference. The endogenous gene Glyceraldehyde-3-phosphate dehydrogenase (GAPDH) was used to normalize the results.

### 3.9. Antioxidant Activity

#### 3.9.1. DPPH Radical Scavenging Activity Assessment

The antioxidant capacity of EAPG and its formulation was evaluated using a modified radical scavenging assay based on the protocol by Les et al. (2015) [30]. The assay was performed in a 96-well microplate, with test samples (3.9 µg/mL to 250 µg/mL) and ascorbic acid as a standard prepared at ten-fold lower concentrations (0.391 µg/mL to 25 µg/mL). For each measurement, 100 µL of the standard or sample was combined with 100 µL of 0.2 mM DPPH^•^ (2,2-diphenyl-1-picrylhydrazyl) solution in methanol. The negative control consisted of 100 µL of distilled water and the DPPH^•^ solution. Following 30 min of incubation in the dark at room temperature, the absorbance was measured at 515 nm using a PerkinElmer EnSpire Multimode Plate Reader (PerkinElmer, Waltham, MA, USA). Radical scavenging activity was calculated according to the equation:

% Antioxidant activity = [(C − A)/C] × 100(1)

where C represents the absorbance of the negative control and A represents the absorbance of the sample or standard, both subtracted by their respective blanks.

All samples were analyzed in triplicate across at least two experiments to ensure reproducibility.

#### 3.9.2. Ferric Reducing Antioxidant Power (FRAP) Assay

The reducing capacity of EAPG was evaluated using the FRAP assay, as described by Kelman et al. (2012) [57], with slight modifications. The FRAP working solution was freshly prepared by mixing 300 mM acetate buffer (pH 3.6), 10 mM 2,4,6-tripyridyl-s-triazine (TPTZ) in 40 mM HCl, and 20 mM FeCl_3_.6H_2_O in a 10:1:1 (*v*/*v*/*v*) ratio, which was preheated to 37 °C before use. For the assay, 20 µL of sample or ascorbic acid standard was combined with 150 µL of the FRAP working solution in a 96-well plate. Following an 8 min incubation at room temperature in the dark, absorbance was measured at 595 nm using a multimode plate reader (EnSpire, PerkinElmer, Singapore). Appropriate controls were included by measuring the absorbance of the sample with FRAP solution lacking TPTZ. Quantification was achieved using a standard curve generated with FeSO_4_.7H_2_O (10–350 μM), and results were expressed as μM Fe^2+^ equivalents. All measurements were performed in triplicate across at least two independent experiments to ensure reliability.

### 3.10. Cytotoxicity Assessment of EAPG on hGF Cells Using MTT Assay

Cells were seeded at a density of 1 × 10^3^ cells/well in 96-well plates and allowed to adhere overnight. Following attachment, cells were treated with increasing concentrations of EAPG (0 to 1000 µg/mL) for 24 and 48 h to assess both acute and subacute effects. After treatment, cellular metabolic activity was determined by adding 10 μL of 3-(4,5-dimethylthiazol-2-yl)-2,5-diphenyltetrazolium bromide (MTT) solution (5 mg/mL in PBS) and incubating for 4 h to allow formazan crystal formation. The supernatants were discarded, and 100 μL of acidified isopropanol was added to dissolve the formazan crystals. Absorbance was then measured at 570 nm using a microplate reader Perkin-Elmer EnSpire Multimode Plate Reader, (PerkinElmer, Waltham, MA, USA). As control, untreated hGFC was used to establish baseline cell viability (100%).

### 3.11. Antimicrobial Assays

#### 3.11.1. Yeast Cultivation and Inoculum Preparation

The study utilized reference strains from the American Type Culture Collection (ATCC), including *Candida albicans* (ATCC 90028), *Candida glabrata* (ATCC 90030), and *Candida krusei* (ATCC 6258), which were maintained in the Microbiology and Clinical Immunology Laboratory (LabMic) at the University of Brasília. For inoculum preparation, each Candida strain was subcultured in Sabouraud Dextrose Broth (Acumedia, Sao Paulo, Brazil) supplemented with 20% dextrose and incubated at 35 °C for 24 h. Following growth, a yeast aliquot was suspended in a 0.85% saline solution, and the yeast density was adjusted to a McFarland scale of 0.5, corresponding to 1–5 × 10^6^ CFU/mL (standard solution), using a calibrated turbidimeter. From this standard solution, a working dilution was prepared by diluting this solution 1:10 to achieve 1–5 × 10^5^ CFU/mL, which was used in the 96-well plate.

#### 3.11.2. Antifungal Susceptibility Test of EAPG Against *Candida* Strains

The antifungal activity of EAPG was evaluated against the three ATCC *Candida* strains using a broth microdilution method adapted from NCCLS M27-A2 guidelines [58]. The assay was performed in 96-well microplates. EAPG was tested at concentrations ranging from 0.019 to 10 mg/mL, alongside clinical antifungals as comparators: fluconazole (0.03–64.0 mg/mL), itraconazole (0.004–8.0 mg/mL), and amphotericin B (0.004–8.0 mg/mL).

### 3.12. Development and Characterization of EAPG-Loaded Chitosan Nanoparticles

EAPG was successfully encapsulated into chitosan nanoparticles using an optimized ionotropic gelation method [59]. The process began with dissolving 5 g of chitosan in 1% (*v*/*v*) acetic acid solution (pH 5.0) under continuous magnetic stirring (300 rpm) for 24 h at room temperature. EAPG was then incorporated into a chitosan solution at a concentration of 5 mg/mL, followed by controlled ultrasonication using pulsed cycles (30 s ON/OFF at 30% amplitude) to ensure complete dispersion of extract components while preventing thermal degradation.

For comprehensive characterization, the nanoparticle formulations underwent multiple analytical assessments. The size distribution of the nanoparticles (mean diameter and polydispersity index, PdI) was determined by dynamic light scattering using a Zetasizer Nano ZS (Malvern Co., Malvern, UK), without requiring sample dilution. The Zeta potential was determined by electrophoretic mobility using the same equipment. In this case, 500 µL of the solution containing the nanoparticles was suspended in 500 µL of 1 mM NaCl and taken to the proper device for analysis.

To evaluate the entrapment efficiency of EAPG in chitosan nanoparticles, 2 mL of the nanoparticle dispersion was transferred to disposable ultrafiltration devices (Vivaspin^®^ Sartorius, Göttingen, Germany) and centrifuged at 4000 rpm for 20 min for each sample concentration. Subsequently, a 500 µL aliquot of the lower fraction of the device (filtrate) was added to a test tube, and 500 µL of ultrapure water was added to the test tube. The samples were homogenized and filtered using syringes fitted with hydrophilic membranes with a pore size of 0.45 μm. The resulting solutions were analyzed for the area of peak 1 using the HPLC method described in Section 3.6.2. The proportion of entrapped marker was calculated as the percentage of the marker concentration in the microparticles relative to the initial marker concentration added to prepare the EAPG nanoparticles.

Finally, the morphology of the chitosan nanoparticles, entrapping or not EAPG, was assessed by transmission electron microscopy (TEM). The samples were previously diluted 1:100 (*v*/*v*) in purified water, placed on Formvar-coated copper grids, and dried at room temperature. For contrast enhancement, the grids were stained with 20 µL of a 3% uranyl acetate solution and air-dried for 10 min. The samples were then examined under a transmission electron microscope JEM-1011 (JEOL, Tokyo, Japan) at magnifications of 5000× and 20,000×.

### 3.13. Stability Assessment of EAPG-Loaded Chitosan Nanoparticles

The aqueous dispersion of EAPG-loaded chitosan nanoparticles (n = 3 batches) underwent comprehensive stability testing under two storage conditions: room temperature (25 ± 2 °C) and refrigeration (4 ± 2 °C), both without humidity control, over a 30-day period. Samples were stored in sealed amber flasks to protect against light degradation, with analytical aliquots withdrawn at predetermined intervals (days 1, 7, 15, and 30) for evaluation. The stability study monitored five critical parameters: (i) hydrodynamic diameter, by dynamic light scattering; (ii) polydispersity index (PdI) as a measure of size distribution homogeneity; (iii) zeta potential for surface charge characterization; (iv) maker (peak 1) content, via HPLC-DAD analysis to assess phytochemical stability; and (v) pH of the nanodispersion to monitor potential chemical degradation.

### 3.14. Evaluation of Mucosal Penetration Using Franz Diffusion Cells

The penetration efficiency of EAPG from chitosan nanoparticle formulations was compared to that of an aqueous EAPG solution using an ex vivo porcine oral mucosa model in Franz diffusion cells (n = 6 per group). Since the biological tissue was collected from animals slaughtered for human consumption, approval from a research ethics committee was not required.

Fresh swine oral mucosa specimens, obtained from a local slaughterhouse (Bonasa Alimentos, Brazil), were mounted between donor and receptor compartments, with the epithelial surface facing upward towards the donor chamber containing either: (i) EAPG-loaded chitosan nanoparticles, or (ii) EAPG solution (control). The receptor compartment contained 15 mL of phosphate buffer (pH 7.4), which was maintained in a thermostatically controlled bath at 32 ± 2.0 °C for 12 h with constant magnetic stirring (500 rpm) to simulate physiological conditions. Afterwards, the mucosa fragments were removed from the diffusion cell, and the excess sample on the mucosa was removed with distilled water and application of a paper towel. Finally, the diffusion area of the mucosa (1.77 cm^2^) was fragmented into small pieces, immersed in 5 mL of methanol, and left for 24 h under moderate agitation for active extraction. The solution was filtered through a 0.22 μm membrane and subjected to quantification of the EAPG peak using the previous HPLC-DAD method (Section 3.6.2). The results were expressed as the content of the major EAPG (peak 1) that penetrated the mucosa.

### 3.15. Statistical Analysis

All experiments were performed in triplicate (except for the ex vivo mucosal penetration studies, which were conducted in sextuplicate), and data are expressed as the mean ± standard deviation (SD). The Shapiro–Wilk test was applied to assess data normality. Kruskal–Wallis test was used for comparisons including three or more groups. Differences were considered statistically significant at *p* < 0.05. All analyses were performed using GraphPad Prism, version 8.0.2 (GraphPad Software Inc., San Diego, CA, USA).

## 4. Conclusions

This study successfully developed a standardized protocol for producing aqueous extracts from *Psidium guajava* leaves (EAPG), with four production batches demonstrating excellent reproducibility in key quality parameters, including total solid content, yield, moisture content, and total polyphenol content. The EAPG showed significant therapeutic potential for treating oral ulcerations and infections due to its demonstrated antioxidant, anti-inflammatory, and antimicrobial properties. A chitosan-based nanoparticle delivery system was produced, showing high encapsulation efficiency, optimal physicochemical properties, and excellent stability. The nanoparticle formulation significantly enhanced mucosal delivery, increasing tissue retention of active compounds compared to the free extract control, while preventing systemic absorption. These findings position the EAPG-loaded chitosan nanoparticles as a promising therapeutic option for oral mucosal disorders, combining the benefits of traditional herbal medicine with advanced drug delivery technology to address conditions like oral ulcers, candidiasis, and mucositis. The system-enhanced bioavailability, targeted delivery, and phytochemical protection represent a significant advance in herbal medicine formulations for oral healthcare applications.

## Figures and Tables

**Table 1:** Results of the stability test of the aqueous dispersion of chitosan nanoparticles loaded with *Psidium guajava* L. aqueous extract (EAPG) at room temperature.

Room Temperature (25 ± 2 °C)					
Days	Size (nm)	PdI	Zeta Potential (mV)	pH	EE (%)
1	899.83 ± 10.85	0.224 ± 0.03	32.4 ± 2.33	5.0	62 ± 1
2	847.03 ± 4.18	0.289 ± 0.01	32.7 ± 2.16	5.0	nd
7	789.07 ± 21.71	0.312 ± 0.02	33.9 ± 2.37	5.0	nd
15	754.43 ± 5.02	0.387 ± 0.04	30.4 ± 1.89	4.9	65 ± 2
30	711.10 ± 11.26	0.372 ± 0.04	28.5 ± 2.67	4.6	62 ± 3

EAPG: *Psidium guajava* L. aqueous extract; EE%: entrapment efficiency; nd: not determined due to technical issues during the experiment.

**Table 2:** Results of the stability test of the aqueous dispersion of chitosan nanoparticles loaded with EAPG at 4 ± 2 °C.

Temperature 4 ± 2 °C					
Days	Size (nm)	PdI	Zeta Potential (mV)	pH	EE (%)
1	899.83 ± 10.85	0.224 ± 0.03	32.4 ± 2.52	5.0	62 ± 2
2	886.80 ± 8.27	0.297 ± 0.02	31.7 ± 1.85	5.0	62 ± 4
7	894.13 ± 13.90	0.354 ± 0.05	31.4 ± 2.55	5.0	60 ± 2
15	845.40 ± 48.53	0.284 ± 0.04	34.3 ± 2.49	4.9	58 ± 2
30	860.94 ± 44.57	0.292 ± 0.01	32.4 ± 1.92	5.0	68 ± 3

EAPG: *Psidium guajava* L. aqueous extract; EE%: entrapment efficiency.

## Data Availability

The original contributions presented in this study are included in the article/Supplementary Materials. Further inquiries can be directed to the corresponding author.

## Supplementary Materials

The following supporting information can be downloaded at: https://www.mdpi.com/article/10.3390/plants14193099/s1, File S1: HPLC-DAD chromatograms from guaijaverin, hyperoside and isoquercitrin.

## Abbreviations

The following abbreviations are used in this manuscript:

PdI	polydispersity index
EAPG	aqueous extracts of *P. guajava* leaves
TLC	Thin-layer chromatography
HPLC	High-performance liquid chromatography
DAD	Diode Array Detector
NF-kB	Factor nuclear kappa B
LPS	Lipopolysaccharide
hGFC	Hepatocyte growth factor
IL-6	Interleukin 6
MIC	Minimum Inhibitory Concentration
ATCC	American Type Culture Collection
TEM	Transmission electron microscopy
DMEM	Dulbecco’s modified eagle medium
MTT	(3,[4,5-dimethylthiazol-2-yl]-2,5-diphenyltetrazolium bromide)
EDTA	2,2′,2″,2‴-(ethane-1,2-diyldinitrilo) tetraacetic acid
STPP	Sodium Tripolyphosphate
ANVISA	Brazilian Health Regulatory Agency
GAPDH	Glyceraldehyde-3-phosphate dehydrogenase
DPPH	2,2-Diphenyl-1-picrylhydrazyl radical
FRAP	Ferric Reducing Antioxidant Power
