# Supplementary material for: Chitosan Nanoparticles Entrapping Aqueous Psidium guajava L. Leaf Extracts: A Promising Approach for Topically Treating Disorders in Oral Mucosa
Source: Plants (Basel). 2025 Oct 8;14(19):3099. doi: 10.3390/plants14193099 (PMC12526543; doi:10.3390/plants14193099)
Supplement: Supplementary file 1 [file plants-14-03099-s001.zip › plants-3827946-supplementary.pdf]

External Standard Report

Method Name: C:\EZChrom Elite\Enterprise\Projects\Yris Pérola\Métodos\Método_5A_Integração_354nm_nv.met
Data: C:\EZChrom Elite\Enterprise\Projects\Yris Pérola\Data\04092025\Hiperosideo_1mg_mL.dat

User: Controle
Acquired: 04/09/2025 16:39:24
Printed: 05/09/2025 09:23:27

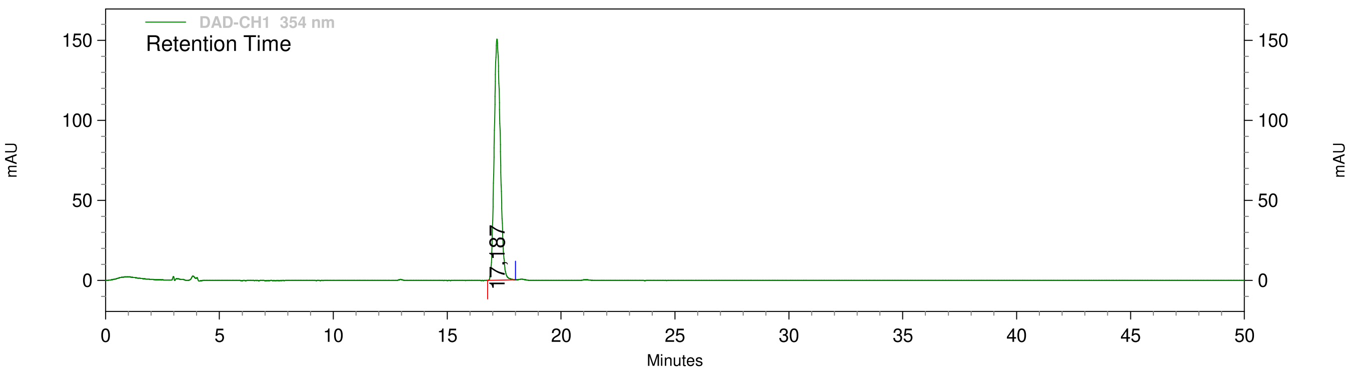

DAD-CH1 354 nm Results

	Area	Retention Time
	11410386	17,187
PermeaPer		

Spectrum Report

Spectra of all detected peaks

(The peak spectrum is defined as the peak apex spectrum)

DAD-CH1 354 nm Spectra

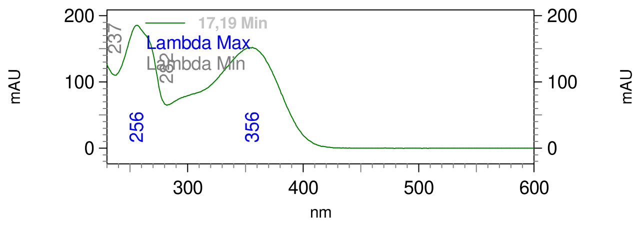

Retention time: 17,187 Min
Peak name:
Lambda max: 256, 356
Lambda min: 282, 237
Purity: 1,000000

Library Search Report

Libraries searched for apex spectra of all detected peaks.

Search Parameters

External Standard Report

Method Name: **C:\EZChrom Elite\Enterprise\Projects\Yris Pérola\Métodos\Método_5A_Integração_354nm_nv.met**
Data: **C:\EZChrom Elite\Enterprise\Projects\Yris Pérola\Data\04092025\Hiperosideo_1mg_mL.dat**

User: **Controle**
Acquired: **04/09/2025 16:39:24**
Printed: **05/09/2025 09:23:27**

Wavelength range: 230 - 400 nm
Wavelength step: 1
Max hits: 4
Similarity threshold:

Pre-filters

Retention time range:
Lambda max:
Compound name filter:

Libraries

- C:\EZChrom Elite\Enterprise\Projects\Renata\Library\Isoquercetrina.lib
- C:\EZChrom Elite\Enterprise\Projects\Renata\Library\Guajaverina.lib
- C:\EZChrom Elite\Enterprise\Projects\Renata\Library\Hiperosídeo_100ug_ml.lib
- C:\EZChrom Elite\Enterprise\Projects\Biblioteca de padrões\Isoquercetina_Sigma 17793_Met.Mod.Pg.lib
- C:\EZChrom Elite\Enterprise\Projects\Biblioteca de padrões\Hiperosídeo_HWI_180585_Met.Mod.Pg.pdf.lib

A peak @ 17,19 Min
detected on channel: DAD-CH1 354 nm
Number of hits: 4

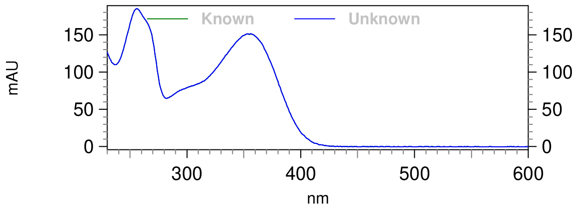

Hit #: 1
Library: C:\EZChrom Elite\Enterprise\Projects\Biblioteca de padrões\Hiperosídeo_HWI_180585_Met.Mod.Pg.pdf.lib
Source: C:\EZChrom Elite\Enterprise\Projects\Yris Pérola\Data\04092025\Hiperosideo_1mg_mL.dat
Component: Spectrum @ 17,18 Min
Retention Time: 17,1800 Min
Similarity: 1,0000

Method Name: C:\EZChrom Elite\Enterprise\Projects\Yris Pérola\Métodos\Método_5A_Integração_354nm_nv.met
Data: C:\EZChrom Elite\Enterprise\Projects\Yris Pérola\Data\04092025\Hiperosideo_1mg_mL.dat

User: Controle
Acquired: 04/09/2025 16:39:24
Printed: 05/09/2025 09:23:27

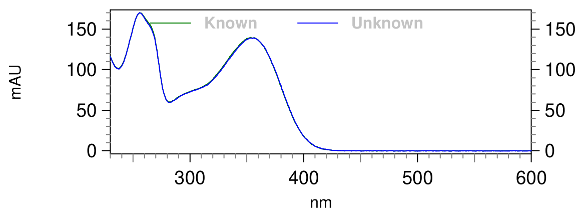

Hit #: 2
Library: C:\EZChrom Elite\Enterprise\Projects\Biblioteca de padrões\Isoquercetina_Sigma 17793_Met.Mod.Pg.lib
Source: C:\EZChrom Elite\Enterprise\Projects\Yris Pérola\Data\04092025\Isoquercitrina_1mg_mL.dat
Component: Spectrum @ 17,72 Min
Retention Time: 17,7200 Min
Similarity: 1,0000

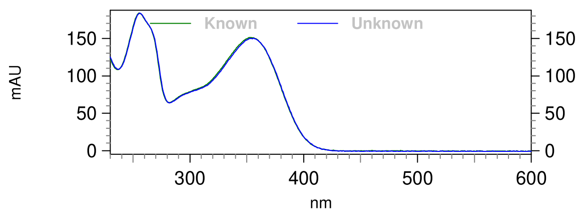

Hit #: 3
Library: C:\EZChrom Elite\Enterprise\Projects\Renata\Library\Hiperosídeo_100ug_ml.lib
Source: C:\EZChrom Elite\Enterprise\Projects\Paula Martins\Funcho\Data\08032022\Hiperosídeo_100ug_ml.dat
Component: Spectrum @ 41,68 Min
Retention Time: 41,6800 Min
Similarity: 0,9999

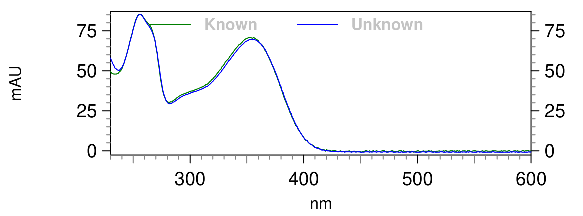

Hit #: 4
Library: C:\EZChrom Elite\Enterprise\Projects\Renata\Library\Isoquercetrina.lib
Source: C:\EZChrom Elite\Enterprise\Projects\Renata\Resultados\04022022_b\Isoquercetina.dat
Component: Spectrum @ 43,26 Min
Retention Time: 43,2600 Min
Similarity: 0,9996

External Standard Report

Method Name: C:\EZChrom Elite\Enterprise\Projects\Yris Pérola\Métodos\Método_5A.met
Data: C:\EZChrom Elite\Enterprise\Projects\Yris Pérola\Data\04092025\Isoquercitrina_1mg_mL.dat
User: Controle
Acquired: 04/09/2025 15:47:55
Printed: 05/09/2025 08:30:25

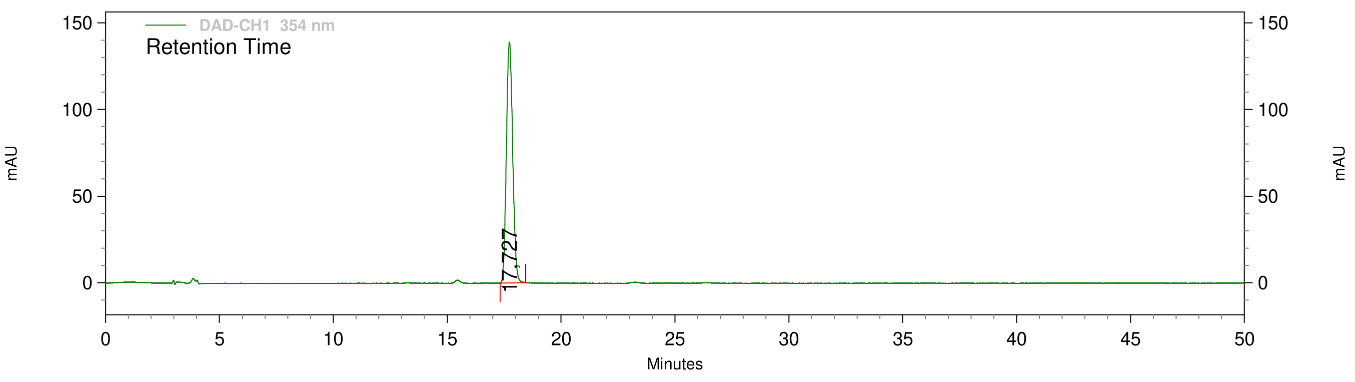

DAD-CH1 354 nm Results

	Area	*Retention Time*
	10710496	17,727

PermeaPer

Spectrum Report

Spectra of all detected peaks

(The peak spectrum is defined as the peak apex spectrum)

DAD-CH1 354 nm Spectra

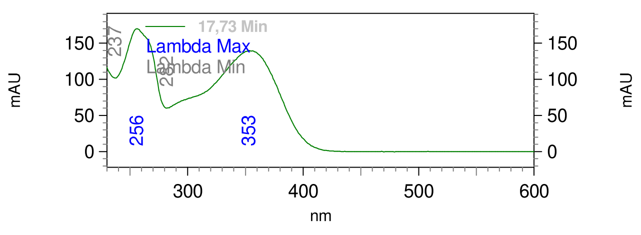

Retention time: 17,727 Min
Peak name:
Lambda max: 256, 353
Lambda min: 282, 237
Purity: 1,000000

Library Search Report

Libraries searched for apex spectra of all detected peaks.

Search Parameters

Wavelength range: 230 - 400 nm
Wavelength step: 1
Max hits: 4
Similarity threshold:

Method Name: **C:\EZChrom Elite\Enterprise\Projects\Yris Pérola\Métodos\Método_5A.met**
Data: **C:\EZChrom Elite\Enterprise\Projects\Yris Pérola\Data\04092025\Isoquercitrina_1mg_mL.dat**
User: **Controle**
Acquired: **04/09/2025 15:47:55**
Printed: **05/09/2025 08:30:25**

Pre-filters

Retention time range:
Lambda max:
Compound name filter:

Libraries

- C:\EZChrom Elite\Enterprise\Projects\Biblioteca de padrões\isoquercitrina 100 µg_mL.lib
- C:\EZChrom Elite\Enterprise\Projects\Biblioteca de padrões\ácido elágico.lib
- C:\EZChrom Elite\Enterprise\Projects\Biblioteca de padrões\hesperetina 14012013.lib
- C:\EZChrom Elite\Enterprise\Projects\Biblioteca de padrões\quercetina.lib
- C:\EZChrom Elite\Enterprise\Projects\Biblioteca de padrões\Hesperidina 14012013.lib
- C:\EZChrom Elite\Enterprise\Projects\Biblioteca de padrões\rutina.lib
- C:\EZChrom Elite\Enterprise\Projects\Biblioteca de padrões\hiperosideo.lib
- C:\EZChrom Elite\Enterprise\Projects\Biblioteca de padrões\Revesratrol.lib
- C:\EZChrom Elite\Enterprise\Projects\Biblioteca de padrões\catequina.lib
- C:\EZChrom Elite\Enterprise\Projects\Biblioteca de padrões\vitexina.lib
- C:\EZChrom Elite\Enterprise\Projects\Biblioteca de padrões\isovitexina.lib
- C:\EZChrom Elite\Enterprise\Projects\Biblioteca de padrões\miricetina 100 µg_mL.lib
- C:\EZChrom Elite\Enterprise\Projects\Biblioteca de padrões\miricitrina 100 µg_mL.lib
- C:\EZChrom Elite\Enterprise\Projects\Biblioteca de padrões\luteolina 11092012.lib
- C:\EZChrom Elite\Enterprise\Projects\Biblioteca de padrões\Ácido cafeico.lib
- C:\EZChrom Elite\Enterprise\Projects\Biblioteca de padrões\Ácido Clorogênico.lib
- C:\EZChrom Elite\Enterprise\Projects\Biblioteca de padrões\Ácido ferúlico.lib
- C:\EZChrom Elite\Enterprise\Projects\Renata\Library\Guajaverina.lib
- C:\EZChrom Elite\Enterprise\Projects\Damaris\biblioteca\cumarina.lib
- C:\EZChrom Elite\Enterprise\Projects\Patricia\Padrões\miricitrina 100 µg_mL.lib
- C:\EZChrom Elite\Enterprise\Projects\Erythroxylum\espectral libery\quercetin.lib

External Standard Report

Method Name: C:\EZChrom Elite\Enterprise\Projects\Yris Pérola\Métodos\Método_5A.met
Data: C:\EZChrom Elite\Enterprise\Projects\Yris Pérola\Data\04092025\Isoquercitrina_1mg_mL.dat
User: Controle
Acquired: 04/09/2025 15:47:55
Printed: 05/09/2025 08:30:25

Following library files cannot be used to search on:

C:\EZChrom Elite\Enterprise\Projects\Damaris\biblioteca\cumarina.lib - file does not exist.
C:\EZChrom Elite\Enterprise\Projects\Patricia\Padrões\miricitrina 100 µg_mL.lib - file does not exist.
C:\EZChrom Elite\Enterprise\Projects\Erythroxylum\espectral libery\quercetin.lib - file does not exist.

A peak @ 17,73 Min
detected on channel: DAD-CH1 354 nm
Number of hits: 4

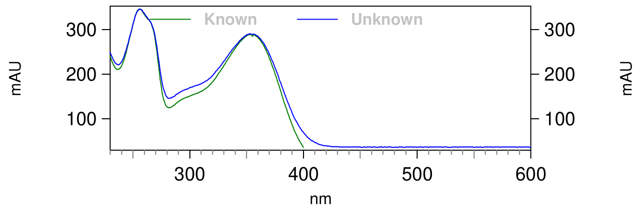

Hit #: 1
Library: C:\EZChrom Elite\Enterprise\Projects\Biblioteca de padrões\rutina.lib
Source: C:\EZChrom Elite\Enterprise\Projects\Damaris\Leonardo\17042013\rutina 1,1mg 3mL.dat
Component: rutina
Retention Time: 27,4000 Min
Similarity: 1,0000

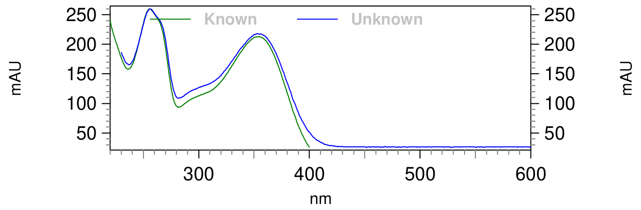

Hit #: 2
Library: C:\EZChrom Elite\Enterprise\Projects\Biblioteca de padrões\isoquercitrina 100 µg_mL.lib
Source: C:\EZChrom Elite\Enterprise\Projects\Patricia\08052015\isoquercitrina 100 ug_mL-Rep3.dat
Component: isoquercitrina 100 ug_mL
Retention Time: 28,6800 Min
Similarity: 0,9999

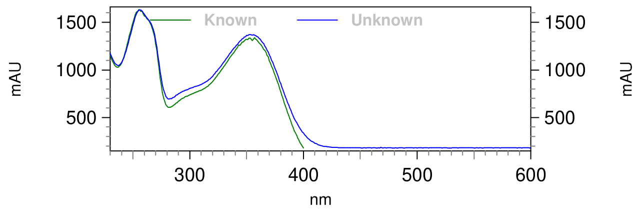

Hit #: 3
Library: C:\EZChrom Elite\Enterprise\Projects\Biblioteca de padrões\hiperosideo.lib
Source: C:\EZChrom Elite\Enterprise\Projects\Damaris\Leonardo\17042013\hiperosideo 1mg 2mL.dat
Component: hiperosídeo
Retention Time: 28,7000 Min
Similarity: 0,9998

External Standard Report

Method Name: **C:\EZChrom Elite\Enterprise\Projects\Yris Pérola\Métodos\Método_5A.met**
Data: **C:\EZChrom Elite\Enterprise\Projects\Yris Pérola\Data\04092025\Isoquercitrina_1mg_mL.dat**
User: **Controle**
Acquired: **04/09/2025 15:47:55**
Printed: **05/09/2025 08:30:25**

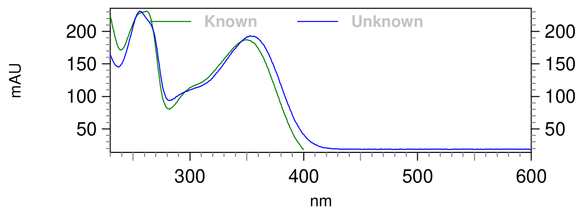

Hit #: 4
Library: C:\EZChrom Elite\Enterprise\Projects\Biblioteca de padrões\miricitrina 100 µg_mL.lib
Source: C:\EZChrom Elite\Enterprise\Projects\Patricia\07052015\Miricitrina.dat
Component: miricitrina 100 µg_mL
Retention Time: 27,3200 Min
Similarity: 0,9939

External Standard Report

Method Name: **C:\EZChrom Elite\Enterprise\Projects\Yris Pérola\Métodos\Método_5A_Integração_354nm.met**
Data: **C:\EZChrom Elite\Enterprise\Projects\Yris Pérola\Data\05092025\Pguajava.dat**
User: **Controle**
Acquired: **05/09/2025 14:27:37**
Printed: **16/09/2025 14:20:40**

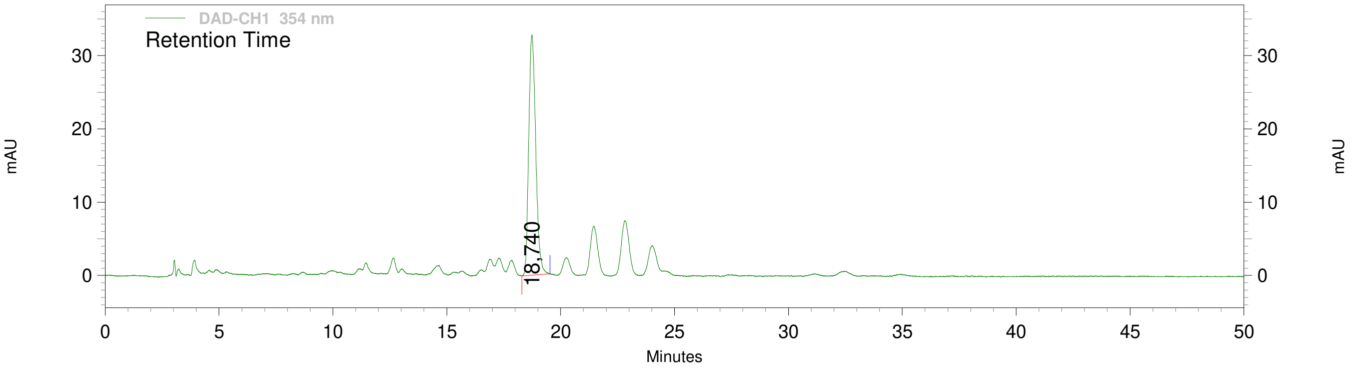

DAD-CH1 354 nm Results

	Area	***Retention Time***
	2816156	18,740

PermeaPer

Spectrum Report

Spectra of all detected peaks

(The peak spectrum is defined as the peak apex spectrum)

DAD-CH1 354 nm Spectra

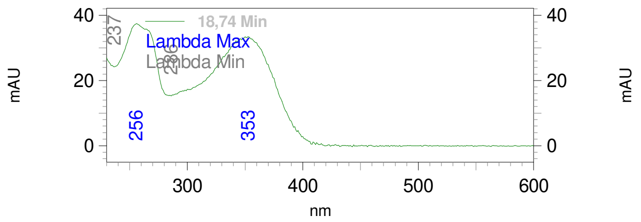

Retention time: 18,740 Min
Peak name:
Lambda max: 256, 353
Lambda min: 286, 237
Purity: 1,000000

Library Search Report

Libraries searched for apex spectra of all detected peaks.

Search Parameters

Wavelength range: 230 - 400 nm
Wavelength step: 1
Max hits: 4
Similarity threshold:

Method Name: **C:\EZChrom Elite\Enterprise\Projects\Yris Pérola\Métodos\Método_5A_Integração_354nm.met**
Data: **C:\EZChrom Elite\Enterprise\Projects\Yris Pérola\Data\05092025\Pguajava.dat**
User: **Controle**
Acquired: **05/09/2025 14:27:37**
Printed: **16/09/2025 14:20:40**

Pre-filters

Retention time range:
Lambda max:
Compound name filter:

Libraries

- C:\EZChrom Elite\Enterprise\Projects\Renata\Library\Guaijaverina_13maio22.lib
- C:\EZChrom Elite\Enterprise\Projects\Biblioteca de padrões\Isoquercetina_Sigma 17793_Met.Mod.Pg.lib
- C:\EZChrom Elite\Enterprise\Projects\Biblioteca de padrões\Hiperosídeo_HWI_180585_Met.Mod.Pg.pdf.lib

A peak @ 18,74 Min
detected on channel: DAD-CH1 354 nm
Number of hits: 3

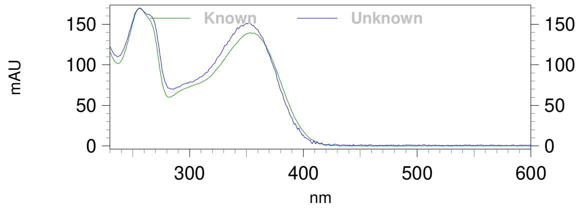

Hit #: 1
Library: C:\EZChrom Elite\Enterprise\Projects\Biblioteca de padrões\Isoquercetina_Sigma 17793_Met.Mod.Pg.lib
Source: C:\EZChrom Elite\Enterprise\Projects\Yris Pérola\Data\04092025\Isoquercitrina_1mg_mL.dat
Component: Spectrum @ 17,72 Min
Retention Time: 17,7200 Min
Similarity: 0,9979

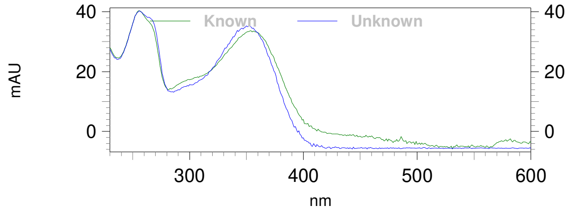

Hit #: 2
Library: C:\EZChrom Elite\Enterprise\Projects\Renata\Library\Guaijaverina_13maio22.lib
Source: C:\EZChrom Elite\Enterprise\Projects\Renata\Maio_2022\Resultados\11052022\Guaijaverina.dat
Component: Spectrum @ 20,71 Min
Retention Time: 20,7067 Min
Similarity: 0,9978

External Standard Report

Method Name: **C:\EZChrom Elite\Enterprise\Projects\Yris Pérola\Métodos\Método_5A_Integração_354nm.met**
Data: **C:\EZChrom Elite\Enterprise\Projects\Yris Pérola\Data\05092025\Pguajava.dat**
User: **Controle**
Acquired: **05/09/2025 14:27:37**
Printed: **16/09/2025 14:20:40**

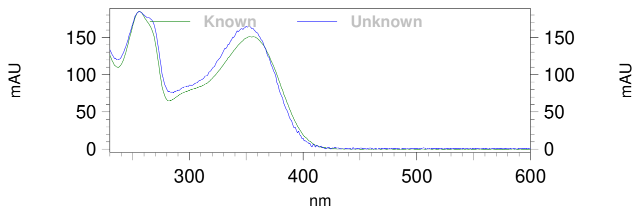

Hit #: 3
Library: C:\EZChrom Elite\Enterprise\Projects\Biblioteca de padrões\Hiperosídeo_HWI_180585_Met.Mod.Pg.pdf .lib
Source: C:\EZChrom Elite\Enterprise\Projects\Yris Pérola\Data\04092025\Hiperosideo_1mg_mL.dat
Component: Spectrum @ 17,18 Min
Retention Time: 17,1800 Min
Similarity: 0,9975
